# Parental Satisfaction with the Quality of Care in an Early Intervention Service for Children with Visual Impairment: A Retrospective Longitudinal Study

**DOI:** 10.3390/children11020230

**Published:** 2024-02-10

**Authors:** Tiziana Battistin, Elena Mercuriali, Carlotta Borghini, Maria Eleonora Reffo, Agnese Suppiej

**Affiliations:** 1 Department of Neurosciences and Rehabilitation, University of Ferrara, 44121 Ferrara, Italy; 2 Robert Hollman Foundation, 35143 Padova, Italy; e.mercuriali@fondazioneroberthollman.it (E.M.); me.reffo@fondazioneroberthollman.it (M.E.R.); agnese.suppiej@unife.it (A.S.); 3 Robert Hollman Foundation, 28821 Cannero Riviera, Italy; 4 Unit of Biostatistics, Epidemiology and Public Health, Department of Cardiac, Thoracic, Vascular Sciences and Public Health, University of Padua, 35131 Padua, Italy; carlotta.borghini@ubep.unipd.it; 5 Department of Medical Sciences, University of Ferrara, 44121 Ferrara, Italy

**Keywords:** early intervention, pediatrics, infants, vision, low, blindness, parents, interpersonal skills, quality of care, caregivers, rehabilitation

## Abstract

The fundamental role of vision during development and the nurturing role of early intersubjectivity have enabled the Robert Hollman Foundation to develop an early intervention program providing holistic support to visually impaired children and their families, where fostering parent-infant interactions is at the heart of our care. The aim of this study is to understand how parents perceive this approach. It is an eleven-year retrospective study of children following the Robert Hollman Foundation’s early intervention program, in which parents’ (*n* = 1086) perceptions of quality of care were measured through the administration of a specifically designed 4-point scale questionnaire. Annual longitudinal trends of parents’ perceptions were calculated for every single response. Parents reported a very high satisfaction value in 21/23 questions (Mean > 3.7 out of a maximum score of 4, with the highest scores in human and soft skills of professionals) with a statistically positive trend (*p* < 0.05), throughout the period considered. Our core approach, based on an *individualized nurturing relational support*, has been appreciated and confirmed by the high satisfaction reported in the questionnaires by parents of children with visual impairment. We therefore hypothesize that parent-infant relationship-based and individualized approaches may help parents achieve better health, well-being, and quality of daily life for their children.

## 1. Introduction

Recent data from the Global Vision Database Report estimated that 22 million children have a medium-severe visual impairment (VI), 40 million a mild VI and 1.4 million are blind worldwide [1]. The majority of them present a congenital or early onset VI [2,3], which is an added risk factor for dysfunction in all of the developmental functions, particularly attachment, sleep-wake cycle, learning, gross and fine motor ability, communicative skills and cognitive ability [3,4,5,6,7,8,9] and also for developmental setback [10,11].

Indeed, vision plays a fundamental role in child development [12,13] and has a scaffolding function for social behaviour [14]. Research has shown that visual function is the “synthesizer of experience” ([9], p. 115) [13,15], coordinating all other sensory systems and guiding interaction with the external world [16]. Rudimentary visual pathways are already present at birth, but their maturation is a long process, strongly influenced by experience-dependent plasticity during critical periods in the early years of life [17]. Children’s early years are crucial to their development as they represent a relentless exploration of and interaction with the world around them, leading to continuous discovery and learning. Daily interaction with their surroundings, starting with the faces of their caregivers and subsequently objects/people/situations, enables the development of affective, communicative, cognitive, motor and social skills, which continue to be constantly supported by vision [4]. Consequently, VI is a main issue in health policy, impacting profoundly children’s development and quality of life [18]: its global burden is associated with lower socio-economic status and mental health, reduced educational and employment opportunities, as well as with an increased risk of death [19,20]. It can also have a more devastating impact in young children than in adults due to its long-life duration [21]. In a recent paper, a decrease in global burden of VI has been reported in paediatric age, which may be attributed to the advances in timely identification of visual deficit and early intervention [22].

The literature suggests that the crucial role of early intervention as “neuroprotective” strategy, which stimulates brain development, particularly during the window of maximal plasticity to foster the future development and quality of life of the child [23,24]. A primary aim of early intervention is to offer socio-emotional support and education to the child’s caregivers in order to enhance their competence and confidence in their role to favour the expression of child’s maximum potential. Parent-infant intersubjective interaction is the first natural environment in which the early intervention can take place [25], so family has a fundamental role being an active participant and its satisfaction and perceptions are important indicators of the quality of care of the early intervention received [26,27].

Despite the great number of articles on early intervention in literature, there are still relatively few recent contributions regarding early intervention programs in infants with visual impairment, as reported in recent reviews, aimed at evaluating their effectiveness [28,29]. The parents’ satisfaction on the quality of the care received in early intervention is even less studied [3,26,30,31]; although different settings and populations were studied, the quality of the family’s support played an important role in all studies.

The Robert Hollman Foundation (RHF) has been developing, since 1979, a holistic early intervention program with the aim of supporting the development of children with congenital/early onset visual impairment through a relationship-based approach in a specially designed environment. The conceptual roots of this program are the psychological theories on the affective-relational family development [32,33,34] and results of research on neuro-visual interplay during development occurring in children with vision loss in the first months of life [15,35]. These foundations, combined with the long-lasting experience of RHF professionals in their daily work with children with VI and with their families, allowed the implementation of this early intervention program with an individualized nurturing support to the family nucleus [36,37,38,39]. There are no studies that have evaluated how programs with these characteristics are viewed and whether they are appreciated by the families.

Among the tools evaluating the quality of care in paediatric intervention services, the Measure of Processes of Care (MPOC-20) has been designed to measure parents’ perceptions of the care they and their children with disabilities receive from specialized rehabilitation centres, covering all of the dimensions of caregiving, interpersonal aspects of care and informational needs, believing that the interactions between professionals and parents may have an effect on parental well-being [40]. This questionnaire, however, is not focused on the special population of children with visual impairment and on a structure such as the RHF. Consequently, fourteen years ago, mirroring the MPOC-20, the RHF developed a specific questionnaire in order to assess the satisfaction of the families of children with visual impairment undergoing a residential early intervention program at the Foundation.

In this study, we retrospectively reviewed the questionnaires collected at the RHF with the aim of assessing parents’ satisfaction on our residential early intervention program that emphasizes caring and nurturing relationships for better health and provides individualized holistic support for the development of the child with visual impairment.

## 2. Materials and Methods

### 2.1. Study Design

This study has a retrospective longitudinal design, which took place at the RHF, a no-profit institution, specifically devoted to supporting the development of children with low vision or blindness, and their families. We selected parents of all children in the age range of 0–4 years who performed the same residential early intervention program, in order to obtain homogeneous data.

This study considered questionnaires provided by parents over an 11-year period (from January 2009 to February 2020 until closure due to the COVID-19 pandemic).

The following formula was considered for estimating the sample size:

$$\mathrm{precision}\;=2\times Z_{\frac{\alpha }{2}}\frac{\sigma }{\sqrt{n}}.$$

We began by defining precision, that is, the width of the confidence interval around the estimated mean. As parameters, we assumed a precision of 0.15, a conservative standard deviation of 1, and a confidence level of 95%. Using these parameters, a sample size of approximately 685 questionnaires was obtained. The conducted collection, which consisted of 1081 questionnaires, allowed for maintaining the desired precision.

The research was carried out in accordance with the Declaration of Helsinki guidelines of the World Medical Association [41].

### 2.2. Participants

One thousand and eighty-six questionnaires were completed by parents of children with congenital or early onset vision loss defined according to Italian law (no. 183/2001) as visual acuity less than 3/10 and/or a visual field defect of less than 60% in the better eye.

### 2.3. Early Intervention Program Assessed by Questionnaires

Fundamental to RHF’s early intervention program, evaluated by these questionnaires, is an *individualized nurturing support* in which the relationship between the child, their families and the health professionals is integrated, permeates and shapes the evaluation process, not only of visual function and functional vision, but also of all areas of development. The duration of this early intervention program ranges from one to three weeks, during which the parents live with their child at the Foundation where they undergo a multidisciplinary assessment and habilitation program. It starts with a preliminary evaluation of medical records and a subsequent comprehensive observation of the child’s global and visual functioning carried out by a multi and inter-disciplinary team, coordinated by a psychologist. It includes a Functional Visual Assessment with the evaluation of visual characteristics and visual behaviors from a multisensory and neuropsychomotor integration perspective. It comprehends evaluation of environmental situations and materials that favour the activation of the different senses, identification of the most appropriate postures to promote sensory attention and finally, careful assessment of how the visual impairment affects relational-affective development. After few a days of assessment, the child and their family begin to be engaged in various activities: neuro-visual and psychomotor habilitation, educational activities, family support, parent training for visual impairment, music therapy, orientation and mobility/Braille pre-requisites. Neuro-visual habilitation has the aim to strengthen the visual functions and functional vision, through multisensory inputs (such as toys/objects with accentuated visual, sound and tactile characteristics), an adapted environment with visual facilitations (such as increasing the contrast figure/background, regulating the illumination or illuminating toys, regulating the distance and position of target presentation) in an interactive and parent-mediated modality. Psychomotor habilitation has the objective of sustaining child development through body play and tonic emotional dialogue, considering movement always inside a relation. Educational activities focus on multisensory integration and activities of daily living and foster visual and overall development, through play. Family support and parent training for visual impairment help parents to face the difficulties and challenges consequent to the birth of their child, introducing them gradually to the knowledge of what is visual impairment and what are the consequences in all of the aspects of daily life, sustaining at the same time their fundamental role as parents. Music therapy enables parents to tune with their baby/child through sound, melodies and also body sounds. Orientation and mobility activities focus on promoting first the ability of localizing, reaching and grasping an object after hearing a sound input, known as “Reach on sound” [42] and then the navigation in space with the aid of tactile and sound cues or with the introduction, when necessary, and preliminary training to the use of the white cane. Braille pre-requisites consist in all those activities for preschool children, aimed at the acquisition of spatial concepts and of the Braille code. These activities are carried out, both individually and in groups with other children/families (six-seven) attending the same early intervention program. Parents are active participants in these activities, observing and interacting both with their children, the professionals and the other families. During the last day, the multidisciplinary team shares with the parents the program’s outcomes such as the child’s new learning, their strengths as well as actual needs. This final restitution meeting allows for reflection on future strategies to promote the child’s development and wellbeing in their area of residence together with local health-care services.

The setting is carefully adapted for each individual family, by planning the space (both environmental structure, activities and relational space) and the time (based on the slow health approach). In addition, emphatic and active listening, a supporting and respectful hospitality and care occur throughout the whole intervention. Constantly attentive, participating and devoted observation helps the professionals to collect even minimally useful details associated with a continuous search for facilitating the expression of the child’s and family’s abilities. Finally, the reassurance of being co-builders in their child’s development, in the respect of the different roles, helps the parents to feel that they are the main experts of their child [43] and to trust the build-up of a family-RHF-local health services network. During the period of this study, the early intervention program performed by children whose parents completed the questionnaire was not modified substantially, except for the duration of the stay, which was shortened from three to two weeks approximately.

### 2.4. The RHF Questionnaire

The questionnaire was given to parents the last day of the stay. It was devised by the RHF health professionals and was based on the MPOC-20 [40] and adapted to account for the particular type of disability that is visual impairment and to consider the need to measure the relationship-based core of this early intervention program.

It uses a 4-point Likert-type scale with responses ranging from “Very unsatisfied” (1) to “Very satisfied” (4).

The questionnaire consists of 23 questions covering three main areas:

The first area is composed of 13 questions focused on services provided in this early intervention program:

- Information given by the RHF at the arrival (1 question)
- Human and soft skills of the team (5 questions)
- Help and support received (1 question)
- Technical skills of the team (2 questions)
- Involvement of parents as active participants (1 questions)
- Scheduling/planning of the activities (2 questions)
- RHF availability of collaboration/work in network with other health services (1 question)

The second area is composed of 6 questions focused on medical consultancies:

- Technical skill of the physician (2 questions)
- Human and soft skill of the physician (2 questions)
- Parents participation to the evaluations (1 question)
- Time dedicated to parents (1 question)

The third area is composed of 4 questions focused on the hosting:

- Behaviour of the personnel regarding hosting and needs (2 questions)
- Comfort of the structure (1 question)
- Hygiene of the structure (1 question)

(See Supplementary Materials for detailed descriptions of the questions).

The questionnaire was completed anonymously, and no other information was included, to allow parents to freely express themselves and make comments regarding their perceptions of the quality of care, stay and the care facilities.

### 2.5. Statistical Analysis

Descriptive statistics, reported as mean (standard deviation), were utilized. For each specific question, a univariate linear model was constructed to evaluate changes in average satisfaction over time. In this context, the beta coefficient is interpreted as the mean change in the score for a given question with each passing year. Specifically, a positive beta implies a positive trend, indicating a mean score increase over the years, while a negative beta suggests a negative trend. The presence of some unanswered questions (missing data) led to the decision to model individual questions rather than aggregating results score by areas, as this would have resulted in skewed scores due to the presence of missing data. The complete results, including beta coefficients for predictors, confidence intervals, and *p*-values, can be visualized in the supplementary materials. All analyses were carried out using the R statistical software (Version 4.3.0) [44].

## 3. Results

In general, the results of the questionnaires showed a very high satisfaction value; all of them scored >3.6 out of a maximum score of 4. The mean value in the first area “Services provided in the early intervention program” was 3.82 (SD = 0.40); in the second area of medical consultancies the mean was 3.78 (SD = 0.44) and finally in the third area of hosting the mean was 3.83 (SD = 0.38).

**Figure 1:**
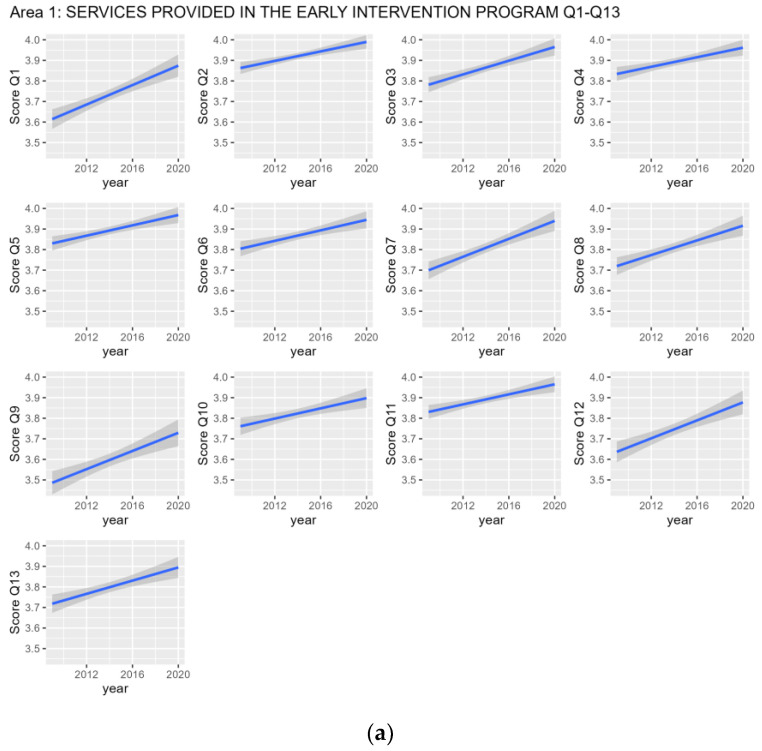

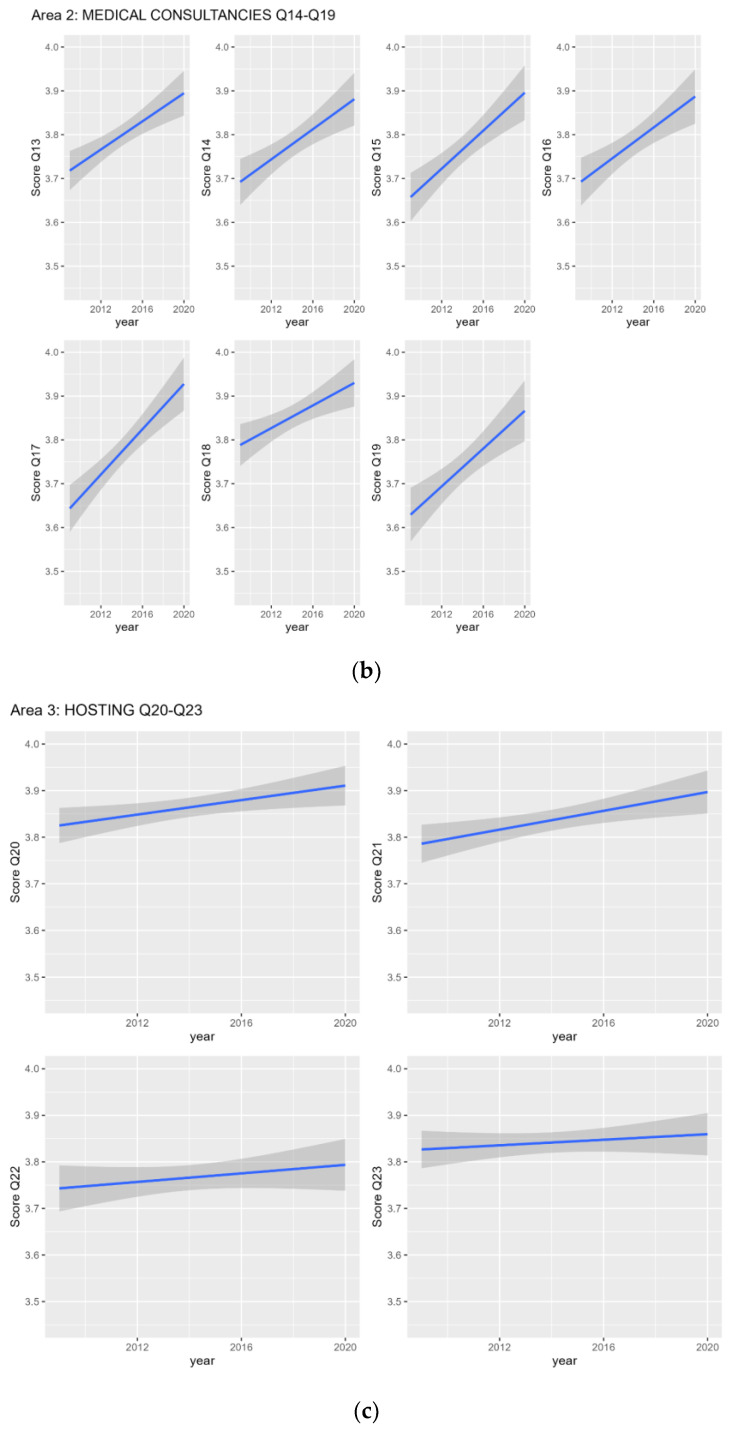
The trend of parental satisfaction estimated by Linear Models for each individual question in the questionnaire over the period 2009–2020, in particular the panel (**a**) for the questions Q1–Q13 of the first area, the panel (**b**) for the questions Q14–Q19 of the second area and the panel (**c**) for the questions Q20–Q23 of the third area. The gray areas around the graph blue lines correspond to the 95% confidence interval.

Parents’ perceptions to all questions (Q1-Q21) showed very high mean scores, regardless of year, and a statistically significant positive trend from 2009 to February 2020, but two (item Q22 and Q23), as reported in Table 1 and Figure 1.

The highest scores were found in the questions related to human skills and soft skills (see Table 1).

## 4. Discussion

The results of this study suggest that an early intervention program, which emphasizes caring and nurturing relationships for better health, is highly appreciated by the families of young children with visual impairments.

We found an overall general satisfaction and appreciation by the parents, with very high mean scores in any single question and particularly in some items of the first area regarding the services provided in this early intervention program. Data from the literature regarding parents’ perceptions in rehabilitation and early intervention services report that families valued and scored higher for respectful and supportive care, regardless of children’s type of disability [27,45,46,47,48]. Only a few studies evaluated parents’ satisfaction in early intervention programs with children with VI [3,26,30,31]. Dale et al. [3] showed an improved satisfaction with parent-practitioner partnership in a home-based early intervention in children with severe visual impairment, which used a resource based on an individualized and family-centred-care approach. Neofotistou et al. [26] evaluated the early intervention program adopted in Greece for children with visual impairment in multiple disabilities; being responsive to child and family needs, support, information and guidance to parents was associated with high scores. Sarimski et al. evaluated early intervention services for children with different types of disability including six in Germany; they showed that more than 30% of the parents were only partially satisfied, especially with the amount and quality of family support [30]. Those properly supported were more satisfied, suggesting an association between support given by professionals and family’s empowerment.

Although the setting as well as the characteristics of the early intervention program of the present study are different from those reported in the literature, all studies confirm the importance for families of human qualities over the technical features and efficiency, and the preference of soft skills, particularly interpersonal, over hard skills in a program of early intervention and developmental support [49].

The literature reports few other early intervention programs for infants with visual impairment [3,13,28,50], which are quite similar to ours for characteristics such as the child and family-centred care approach, with a focus on child’s strengths and needs and a multisensorial, multidimensional and interdisciplinary approach; all of these, except one [3], evaluated their efficacy with other parameters and did not focus on parents’ satisfaction.

We hypothesize that the high satisfaction demonstrated by parents in the questionnaires, is the result of the core approach of the RHF based on two pillars: a *nurturing relational support* and an *individualized approach* to help the parents achieve better health, well-being and quality of daily life for their children and themselves. Indeed, the Hollman early intervention program focuses on the health of the entire affective-relational family nucleus, giving importance to the needs, difficulties, strengths and weaknesses of the entire family, not just the child [37,39]. This hypothesis is in line with a recent study by Chen and Callahan Groves [51], which highlights the importance of emotional support for families of children with visual impairment in early intervention programs and services, showing how relationship-focused early intervention programs have led to positive developmental outcomes for children with disabilities [52].

One possible explanation of the importance of the described approach for the families is that visual impairment, particularly when it occurs early in life, prevents eye contact, a very important foundation for early social and affective development [33]. Thus, *nurturing relational support* is particularly important in the habilitation path of visually impaired children. We propose to consider it as the first pillar in enabling children with VI “to fall in love with the outside world”, which for them is not attainable without adult guidance. The questionnaire was designed to favour the collection of data regarding the evaluation of human and soft skills so it devotes five questions to this aspect. Families gave the highest scores especially to these skills, indicating that they feel actively listened to by all professionals. In fact, *emphatic and active listening* allows the families to open gradually up to dialogue, to share their specific needs, to become aware of them, to understand their child’s strengths and weaknesses and their active role in the child’s development process. Families also scored highly the *supporting and respectful hospitality and care* received from professionals. Respectful and supporting care occurs throughout the entire early intervention program and is not limited to the period of therapies. It is one of the most important interpersonal elements of caregiving [27]. This supporting and respectful care allows professionals to enter gradually into the intimacy of relationships, increasing familiarity with families’ daily life situations and making them feel comfortable at all times while discovering together their child’s abilities and strengths. Parents rated positively the accuracy of the intervention, which is characterized by *constantly attentive, participating and devoted observation* where professionals observe useful details, highlighting resources and potentials together with weaknesses throughout the whole early intervention process, in line with the Reflexive Practice approach [53]. At the same time there is a *continuous search for facilitating*, meaning that professionals work to help the child and their family grow better, by suggesting strategies and adaptations of the home environment and materials to facilitate the expression of the child’s abilities. In the respect of the different roles and according to Brazelton and Sparrow [42], parents are retained as the primary experts on their child, so that they feel *reassured of being co-builders* of their child’s development and become active participants throughout the entire stay.

The second pillar of the early intervention program is the *individualized approach*. Parents responded positively to the *tailored time*, in the sense that even though the habilitation activities are carried out as part of a planned programme, professionals maintain daily flexibility to adapt to children’s needs and what they can accept at that particular time. This tailored time is respectful of parents’ needs and communication with them is undertaken when they are ready to listen. It also allows children and families to feel deeply understood and enables them to express their abilities and strengths fully; it allows the professionals to have greater compliance during observation too. One of the questions, in the medical subsection of the questionnaire, was dedicated to this matter of tailored time, highlighting its perceived importance. Providing appropriate time is in contrast to the modern idea of the Lean approach to health care [54] but in our context it has been fundamental in reducing anxiety, creating better bonding and therapeutic alliances, and ultimately nurturing the child development with a positive global economy.

Parents also appreciated *tailored spaces* for every child and family. Their perceptions showed that they felt at ease being hosted at the RHF; both accommodation and therapy rooms were designed to welcome families. High colour contrast, dimming of lighting, visual-tactile markers at the entrance to every room to aid in distinction and basic interior design are common features of all rooms and favour children’s use of their visual function. Additional furniture and materials are chosen and/or created every day according to their specific needs. Space goes beyond the structural space: there is also a dedicated relational space for welcoming the family, listening to their story and their needs, not only at the beginning but also throughout the whole stay. Activity spaces are also individualized: designed on the child’s peculiarities, prepared before their entrance and modifiable during the activities according to emerging needs to be a welcoming space that promotes their best performance.

Finally, parents appreciated the RHF’s collaboration with local health services, to create *a supportive network* for the family in their home.

The trends for the 2009–2019 period showed a statistically significant increase in all of the items regarding the services provided in the early intervention program (area 1) and the medical consultancies (area 2). During that period some small changes occurred, both in the team of professionals and in structural and organizational aspects but the core of this early intervention program, was maintained. A possible explanation for the improvement of the scores, during the period of the study, is that the skills of the professionals matured with time due to their increased experience and specific soft skills training, they received.

In literature, recent studies showed how early interventions in natural environments, such as families’ home, favour communication with families [55]. The RHF, despite the fact that it is a specialized structure, has characteristics in itself of a natural environment such as a home, allowing the families and their children with VI to be hosted inside it, with the opportunity for the families to share daily time and life experiences with other families, becoming this extra time an additional positive factor for supporting each other. This unique feature enables children with VI and their families to live contemporarily aspects of both “scheduled” habilitation therapies in a specialized structure and the routine/home-based early intervention. Moreover, professionals work interacting with the child and their family as a team, who continuously adapts its planning to needs and priorities of the family nucleus.

The limitations of this study are the following: Firstly, we decided that the questionnaire had to be completed anonymously since we wanted the parents to feel free to answer whatever they wanted and eventually also to add their comments. This choice precluded the collection of demographic and clinical data. Secondly, we dedicated this study to the parents of children with homogeneous ages and early intervention program; therefore, the results cannot be generalized to older ages and to children attending daily clinical care facilities. Moreover, these findings are specific to the early intervention program of the Robert Hollman Foundation and may not be generalizable to other settings or to older children. Finally, we adopted a simple descriptive model that did not allow us to estimate the domains of the latent variable underlying the judgment response for each item, such as for example the Rasch model [56]. It is crucial to note that future analyses will take these models into consideration to broaden our understanding and further refine our conclusions.

Despite the limitations, this study has relevance in clinical practice due to the fact that the parents’ perceptions of the quality of this relationship-focused early intervention program are useful information for all of the health professionals who can modify their programmes, activities, also train their personal skills, if necessary. At the same time, this study highlights the importance of training interpersonal skills and reflective practice of health professionals in order to increase parents’ satisfaction, which is associated to family’s empowerment [54]. Finally, these findings confirm the positive role of an ecological, enriched and facilitating environment with tailored space and time where early child and early parenting intervention are strictly intertwined.

The results of this study also have implications for policy and research, contributing to the current debate on the balance between a Lean approach in health and rehabilitation that condenses space and time, targeted for the sake of profit and efficiency, in contrast with other models of health services where space, time and relationships are adapted to children and family needs, aimed at improving their health and quality of life.

In conclusion, the parents’ responses to a dedicated questionnaire, over an 11-year period, showed positive judgements on the perceived quality of care by parents regarding an early intervention program where relationships permeate and shape soft and hard skills of the professionals with the children’ and families’ needs and skills. It made parents feel supported and reinforced their empowerment. The high scores in the soft skills of the professionals confirm the importance of early relationship-focused interventions. The roots lay on the shaping and therapeutic effect of intersubjectivity both with child and parents, on the reflective approach of the health professionals, with deep knowledge of developmental needs of visually impaired children, and on the nurturing role of a supportive and supported family.

## Figures and Tables

**Table 1:** The table shows mean and standard deviation for each question in the three areas and the results for the implemented univariate linear models. In this context, *mean* denotes the average of all responses to a specific question, irrespective of the year. The *beta coefficient* signifies the average change in score with each passing year.

Area	Question		Mean (SD)	Coefficient (β)
*1. Services provided in the Early intervention program*	Q1	Information to family	3.73 (0.44)	0.024 *
	Q2	Courtesy and attention	3.92 (0.27)	0.012 *
	Q3	Received support	3.87 (0.34)	0.017 *
	Q4	Listening to child	3.89 (0.32)	0.012 *
	Q5	Dialogue availability	3.89 (0.32)	0.013 *
	Q6	Accuracy of intervention	3.87 (0.34)	0.013 *
	Q7	Listening to parents	3.81 (0.40)	0.022 *
	Q8	Explanation of intervention	3.81 (0.40)	0.018 *
	Q9	Planning of activities	3.60 (0.53)	0.022 *
	Q10	Active participation of parents	3.82 (0.39)	0.012 *
	Q11	Respect of the family	3.89 (0.31)	0.012 *
	Q12	Punctuality of professionals	3.75 (0.47)	0.022 *
	Q13	Collaboration with health care services	3.80 (0.41)	0.016 *
*2. Medical consultancies*	Q14	Physician expertise	3.78 (0.43)	0.017 *
	Q15	Diagnosis Communication	3.77 (0.45)	0.022 *
	Q16	Listening skills	3.78 (0.44)	0.018 *
	Q17	Active participation of parents	3.77 (0.44)	0.026 *
	Q18	Courtesy and attention	3.85 (0.38)	0.013 *
	Q19	Dedicated time	3.74 (0.50)	0.022 *
*3. Hosting*	Q20	Hosting	3.86 (0.34)	0.008 **
	Q21	Response to daily needs	3.84 (0.37)	0.010 **
	Q22	Comfort	3.77 (0.44)	0.005
	Q23	Hygiene	3.84 (0.37)	0.003

* *p*-value < 0.001, ** *p*-value < 0.05.

## Data Availability

The data presented in this study are available on request from the corresponding author. The data are not publicly available due to specific ethical and privacy considerations.

## Supplementary Materials

The following supporting information can be downloaded at: https://www.mdpi.com/article/10.3390/children11020230/s1, RHF parents’ questionnaire.
