# Supplementary material for: Parental Satisfaction with the Quality of Care in an Early Intervention Service for Children with Visual Impairment: A Retrospective Longitudinal Study
Source: Children (Basel). 2024 Feb 10;11(2):230. doi: 10.3390/children11020230 (PMC10887141; doi:10.3390/children11020230)
Supplement: Supplementary file 1 [file children-11-00230-s001.zip › children-2814031-supplementary.pdf]

Fondazione Robert Hollman
Consulenza e sostegno allo sviluppo del bambino con deficit visivo

RHF PARENTS' QUESTIONNAIRE

Dear parents,

we kindly ask you to fill out this questionnaire that will allow us to collect your opinion on our work and on RHF services to help us to better respond to your and your childrens' needs.

We kindly ask you to indicate the response which agrees with your opinion.

This questionnaire is anonymous, but if you wish to be recognized or to be heard regarding your indications and/or comments, please feel free to sign it.

Thank you for your cooperation!

Area 1: Services provided in the Early intervention program

With respect to the various interventions proposed how would you describe your level of satisfaction with:

1. The information provided by the Foundation at the time of entry?

- ○ Very unsatisfied
- ○ Not satisfied
- ○ Satisfied
- ○ Very satisfied

---

2. Courtesy and attention of the personnel:

- ○ Very unsatisfied
- ○ Not satisfied
- ○ Satisfied
- ○ Very satisfied

---

3. Help and support received:

- Very unsatisfied
- Not satisfied
- Satisfied
- Very satisfied

---

4. Professionals listening to children’s needs:

- Very unsatisfied
- Not satisfied
- Satisfied
- Very satisfied

---

5. Availability to dialogue

- Very unsatisfied
- Not satisfied
- Satisfied
- Very satisfied

---

6. Accuracy of assessment-rehabilitation interventions

- Very unsatisfied
- Not satisfied
- Satisfied
- Very satisfied

---

7. Professionals listening to parents’ needs

- Very unsatisfied
- Not satisfied
- Satisfied
- Very satisfied

---

8. Explanation of proposed rehabilitation interventions

- Very unsatisfied
- Not satisfied
- Satisfied
- Very satisfied

---

9. Planning of daily activities

- Very unsatisfied
- Not satisfied
- Satisfied
- Very satisfied

---

10. Possibility for parents to participate actively in the child's care-path

- Very unsatisfied
- Not satisfied
- Satisfied
- Very satisfied

---

11. Respect for the individual and their privacy

- Very unsatisfied
- Not satisfied
- Satisfied
- Very satisfied

---

12. Punctuality of professionals

- Very unsatisfied
- Not satisfied
- Satisfied
- Very satisfied

Fondazione Robert Hollman
Consulenza e sostegno allo sviluppo del bambino con deficit visivo

13. Availability of the RHF to collaborate with local health-services

- Very unsatisfied
- Not satisfied
- Satisfied
- Very satisfied

Area 2: Medical consultancies

With respect to medical consultations how would you describe your level of satisfaction with:

14. Professionalism of the physician

- Very unsatisfied
- Not satisfied
- Satisfied
- Very satisfied

15. Communication of the diagnosis, in terms of clarity and comprehensibility

- Very unsatisfied
- Not satisfied
- Satisfied
- Very satisfied

16. Physician's listening to and answering your questions

- Very unsatisfied
- Not satisfied
- Satisfied
- Very satisfied

17. Your involvement in the visits or checks

- Very unsatisfied
- Not satisfied
- Satisfied
- Very satisfied

18. Courtesy and attention

- Very unsatisfied
- Not satisfied
- Satisfied
- Very satisfied

19. Time dedicated to you by the physician

- Very unsatisfied
- Not satisfied
- Satisfied
- Very satisfied

Area 3: Hosting

With respect to the various interventions proposed how would you describe your level of satisfaction with:

20. Welcome of the personnel:

- Very unsatisfied
- Not satisfied
- Satisfied
- Very satisfied

21. Readiness to answer to daily needs:

- Very unsatisfied
- Not satisfied
- Satisfied
- Very satisfied

---

22. Comfort of the building and facilities

- Very unsatisfied
- Not satisfied
- Satisfied
- Very satisfied

---

23. Hygiene of the building and facilities

- Very unsatisfied
- Not satisfied
- Satisfied
- Very satisfied
